# New Operation for Aneurism

**Published:** 1873-11

**Authors:** 


NEW OPERATION FOR ANEURISM. Dr. R. J. Levis, of Philadelphia, recently performed at the Pennsylvania Hospital, an operation for aneurism which is to say the least quite novel in character. He introduced through a fine needle canula several horse-hairs into the sac, with the idea that they would afford sufficient obstacle to the passage of blood to cause coagulation.

We shall look with interest for further reports from the case, and will keep our readers informed concerning it.

